# Erosive tooth wear and salivary parameters among competitive swimmers and non-swimmers in Egypt: a cross-sectional study

**DOI:** 10.1007/s00784-023-05367-7

**Published:** 2023-11-04

**Authors:** Hams H. Abdelrahman, Nour Ammar, Mohamed G Hassan, Wafaa Essam, Hala Amer

**Affiliations:** 1 Department of Pediatric Dentistry and Dental Public Health, Faculty of Dentistry, Alexandria University, Champollion St., Azarita, Alexandria, 21526 Egypt; 2 Department of Conservative Dentistry and Periodontology, University Hospital, Ludwig-Maximilian University of Munich, Munich, Germany; 3 Department of Orthodontics, Faculty of Dentistry, Assiut University, Assiut, Egypt; 4 Division of Bone and Mineral Diseases, Department of Medicine, School of Medicine, Washington University in St. Louis, St. Louis, MO USA

**Keywords:** Erosive tooth wear, Swimmers, Salivary parameters, pH assessment

## Abstract

**Introduction:**

Competitive swimmers spend considerable time practicing their sport. Prolonged exposure to chlorinated water can alter salivary parameters and might compromise oral health. This study aimed to determine erosive tooth wear status and its related risk factors among competitive swimmers as compared to non-swimmers.

**Materials and methods:**

A cross-sectional study consisting of 180 athletes (90 competitive swimmers versus 90 competitive rowers “non-swimmers”) was conducted. Participants were interviewed on the common erosion risk factors. The Basic Erosive Wear Examination system was used to assess the status of erosive tooth wear. Stimulated saliva sample was collected before and after a training session and pool pH was evaluated using pH strips for 7 days. Data were analyzed using descriptive statistics and multivariable analysis.

**Results:**

The prevalence of dental erosion was significantly higher among competitive swimmers (60%) with higher BEWE scores compared to non-swimmers (25.6%). The salivary flow rate was reduced significantly after training sessions in both groups while salivary pH increased among swimmers. Evaluation of pool water revealed a continuous reduction in the pH level, reaching a very acidic pH level of 3.24.

**Conclusion:**

Erosive tooth wear is more prevalent among competitive swimmers. Years of practice and regular consumption of acidic drinks increase the odds of developing erosive lesions. A high incidence of erosive lesions may be attributed to a reduction in swimming pool pH level. Salivary parameters showed variations between groups after training sessions.

## Introduction

Oral health is a vital component of general health and quality of life [1]. Professional athletes need to be well-prepared in order to compete at the highest levels. In that context, poor oral health including dental caries, dental erosive wear, and sports-related oral trauma can compromise an athlete’s performance [2]. In recent years, changes in lifestyle and nutritional habits have contributed to the rise of oral pathologies and dental erosion has become a growing problem, especially among youth [3]. Erosive tooth wear is the chemical loss of mineralized tooth substance caused by exposure to acids not derived from oral bacteria [4]. Several extrinsic factors are involved in the process such as the frequent consumption of acidic foods and beverages and the oral administration of acidic medications [5]. Additionally, environmental acid exposure has been documented as a significant predisposing factor to erosive tooth wear [4].

Swimming is one of the most popular aquatic sports worldwide that has attracted much attention [6]. Professional swimmers undergo a high-intensity training load, spending multiple hours per week in swimming pools [7]. Prolonged exposure to chlorine-based chemicals, such as those used in the disinfection of swimming pools, has a detrimental effect on the swimmers’ hair, skin, and teeth [8, 9]. As a result, the Centers for Disease Control and Prevention (CDC) issued guidelines for Protection Against Recreational Water Illnesses (RWIs) that stipulate that chlorine should be kept at levels between 1.0 and 3.0 ppm, and the pH range should be maintained between 7.2 and 8.0 [10].

Improperly chlorinated swimming pools are often found to have low pH levels, some as low as 3 due to the insufficient buffering of the pool acidity [11]. As a result of the constant exposure to erosive assaults caused by such low pH levels, swimmers’ teeth have become particularly susceptible to enamel erosion, due to the dissolution of hydroxyapatite crystals and the consequent release of calcium ions [12].

Saliva is the critical protective factor in the process of development of erosive lesions. Salivary flow encourages tooth remineralization, provides a buffering effect to resist pH changes, and provides a replenishing supply of minerals to the demineralized tooth surface [13]. However, research established that exercise may have an adverse effect on the flow rate and composition of saliva. Physically active individuals show reduced stimulated salivary flow rates. Consequently, these individuals show a high prevalence of erosive wear lesions, highlighting the association between hard exercise and hyposalivation [14]. Furthermore, the salivary pellicle exhibits anti-erosive properties by acting as a direct barrier that impedes contact between the tooth surface and acids. It also limits the movement of mineral ions, and contains calcium-binding proteins that adjust the concentration of calcium on the tooth surface to a supersaturated level, thus preventing further dissolution of hydroxyapatite [15].

The association between prolonged exposure to pool water and erosive tooth wear has been studied for decades and studies have reported a high prevalence of erosive tooth wear among swimmers [16, 17]. Nevertheless, the integrative effect of all the determinants ought to be thoroughly investigated to further understand any interaction between these factors. Exploring the various factors contributing to the risk of erosive tooth wear among the growing athlete population will eventually help in planning the appropriate management and preventive strategies for this condition.

In view of the evidence showing the increased risk of erosive tooth wear among competitive swimmers and owing to the scarcity of information disclosing this problem in Egypt, the current study aimed to provide a risk assessment profile for competitive swimmers in Alexandria, Egypt. The null hypothesis of this study suggested that there are no significant differences between competitive swimmers and non-swimmer athletes (rowers) regarding erosive tooth wear and salivary parameters.

## Materials and methods

### Study design and ethical approvals

This comparative cross-sectional study recruited a group of competitive swimmers and non-swimmers from public sports clubs in Alexandria, Egypt. Ethical approval was granted by the Ethics Committee of the Faculty of Dentistry at Alexandria University with the number: (IRB 00010556 – IORG 0008839). Informed consent to participate was obtained from the athletes and their legal guardians if they were less than 18 years before recruitment. Additionally, the approval of the directing authorities in the clubs was obtained. All collected saliva samples and associated participant-related data were coded to protect participant confidentiality. The biological samples were safely and permanently disposed of after the completion of the saliva assessment. The examiner provided comprehensive oral hygiene instructions to all participants. The use of a soft-bristled toothbrush and minimally abrasive toothpaste were recommended.

### Participants and eligibility

Competitive swimmers were sampled from the four largest sports clubs in the city. While competitive rowers were selected for the non-swimmer group. Study participants were recruited if they were at least 11 years old, had been training for an average of 7 years or more, and practiced their sport for at least 15 h/week [17]. Conversely, athletes who practiced swimming in addition to rowing and those with systemic diseases affecting salivary flow were excluded. Additionally, informed consent was a prerequisite for participation.

### Sample size estimation

The minimal sample size was calculated based on a study that aimed to analyze the prevalence of erosive tooth wear among competitive swimmers [17]. It was reported that erosive lesions were prevalent in 26% of the competitive swimmers and 10% of the recreational swimmers. Thus, a sample size of 90 participants per group was calculated with 80% power and at a significance level of 0.05 yielding a total sample of 180 athletes. The sample size was calculated using G*Power version 3.1.9.2.

### Reliability and calibration

One examiner (H.A.) performed all the examinations. The intra-examiner reliability was evaluated by clinically examining a group of 20 athletes. The examination was repeated with a week interval and the agreement between the ordinal qualitative data sets from the first and second clinical examinations was assessed using weighted kappa coefficients (K) which resulted in K of 0.807 with a 95% CI (0.69-0.92) which indicated a substantial agreement.

### Data collection

Data collection took place by four methods. The principal investigator (H.A.) approached participants first for an oral examination, and saliva sample collection, followed by an interview, and finally an assessment of the water pH of the swimming pools where the participants trained. Data about demographic information such as age and gender were collected. In addition to sports practice history questions including training hours per day and years of practice.

#### Oral examination

For the oral examination, participants were seated on a chair and the examination was done with the aid of a disposable dental mirror, a source of illumination, and cotton rolls to clean and dry teeth. All permanent teeth surfaces except the third molars were examined for erosive tooth wear lesions using the four-level Basic Erosive Wear Examination (BEWE) while primary teeth were excluded. The most severely affected surface of each sextant was recorded and the cumulative score sum was calculated and transferred into an individual risk level [18] [Additional file 1].

For each participant, two samples were collected. Whole stimulated saliva samples were collected before and after the athletes’ training sessions. Participants were provided with unflavored chewing gum of a standardized size and were instructed to chew on it for 5 min. At 1-min intervals, the participants were requested to provide a saliva sample in pre-labeled graduated falcon tubes. During the process of chewing and sample collection, swallowing was not permitted. The same sampling procedure was repeated immediately after the end of their training sessions [19].

#### Salivary parameters

The salivary rate and pH were determined for each saliva sample. Salivary flow rate (ml/min) was determined by measuring the amount of saliva (ignoring the foam) in milliliters and then dividing it by the collection period in minutes [19]. The salivary pH was assessed using a handheld pH meter (Hl98101 Checker® pH Tester, Hanna Instruments, Italy). Calibration of the pH meter was done before each use by dipping the bulb of the pH meter in a pH 7.0 buffer solution until the buffer was recognized and calibrated automatically as mentioned by the manufacturer’s guidelines. Afterward, the pH electrode was quickly placed in the saliva sample. The pH meter was read and recorded after approximately 10 s once the meter reading was stabilized [20]. Finally, these saliva measurements were compared to reference values for stimulated salivary secretion to detect the salivary status of each patient. Normal pH levels range from 7.2 to 7.5, while normal salivary flow rates range from 1.00 to 3.00 ml/min [21].

Following the completion of the oral examination and the collection of saliva samples, each participant was interviewed regarding the most common risk indicators related to erosive tooth wear that was adopted from the Dental Erosive Wear Risk Assessment form (DEWRA) [21].

#### Swimming pools’ pH level

To ensure a comprehensive assessment of water quality, the pH measurements were conducted daily starting on the day of weekly swimming pool cleaning and chlorination and extending to for a week after. In detail, the pH of each swimming pool was measured twice daily from two different corners of the swimming pool at the start and end of the examination day (averaging about 10 h per day per swimming pool). The second daily water sample was often collected during regular swimming training days when the pool was full of swimmers. This approach allowed us to capture variations in water quality attributable to bathing load.

Competitive swimming necessitates frequent training sessions which leads to prolonged occupancy of the pool by a large number of individuals, resulting in increased water contaminants, and consequently, a greater need to upkeep water chlorination, which in turn results in a drop in pH level as the water is further chlorinated. Maintaining pH at the desired level of 7 is imperative as it serves as a key water quality indicator. This comprehensive sampling strategy allowed us to assess chemical water quality, especially when the swimming pool was fully occupied by swimmers, thereby reflecting the dynamic nature of water quality under different bathing loads.

Swimming pool pH was measured using universal indicator pH strips with a pH range of 6–10 with 0.3 accuracy (Merck KGaA, Darmstadt, Germany) and further confirmed using a digital pH meter [10] [11] [17]. This aimed to provide an overview of the compliance with quality control standards among the included swimming pools in Alexandria, Egypt.

### Outcome assessment

The comparisons between the two groups were deducted by observing the results of the following parameters: scores of the clinical examination using the BEWE scale, measurements of the rate of salivary flow, and pH of whole stimulated saliva. In addition to the assessment of the erosion risk indicators as recorded by the DEWRA form.

### Statistical analysis

The data were analyzed using IBM SPSS, version 23, Armonk, NY, USA. Data were reviewed to check for any errors during data entry. Descriptive statistics were performed using frequencies and percentages for qualitative data while mean and standard deviation (SD) were used for quantitative data. Normality was evaluated using the Kolmogorov-Smirnov test and Q-Q plots. Percentage change in salivary parameters was calculated according to the formula [(after-before)/before] × 100. The differences between both groups were analyzed using the parametric test; Student’s *t*-test and the non-parametric test; the Mann-Whitney *U* test for the quantitative variables. Meanwhile, Pearson’s chi-square test and Fisher’s exact test were used to detect the differences between the qualitative variables. Differences in salivary parameters before and after training sessions were assessed by using paired *t*-tests. Associations between the experience of erosive tooth wear and different risk indicators were tested through a process of univariate logistic regression analysis. Variables with statistically significant relations to the prevalence of erosive tooth wear and some other important factors were introduced into a multivariable logistic regression to create a predictive model for the occurrence of erosive tooth wear. Unadjusted odds ratio (UOR), adjusted odds ratio (AOR), and 95% confidence intervals (CI) were calculated. All tests were two-tailed, and the level of statistical significance was set at 0.05.

## Results

**Fig. 1:**
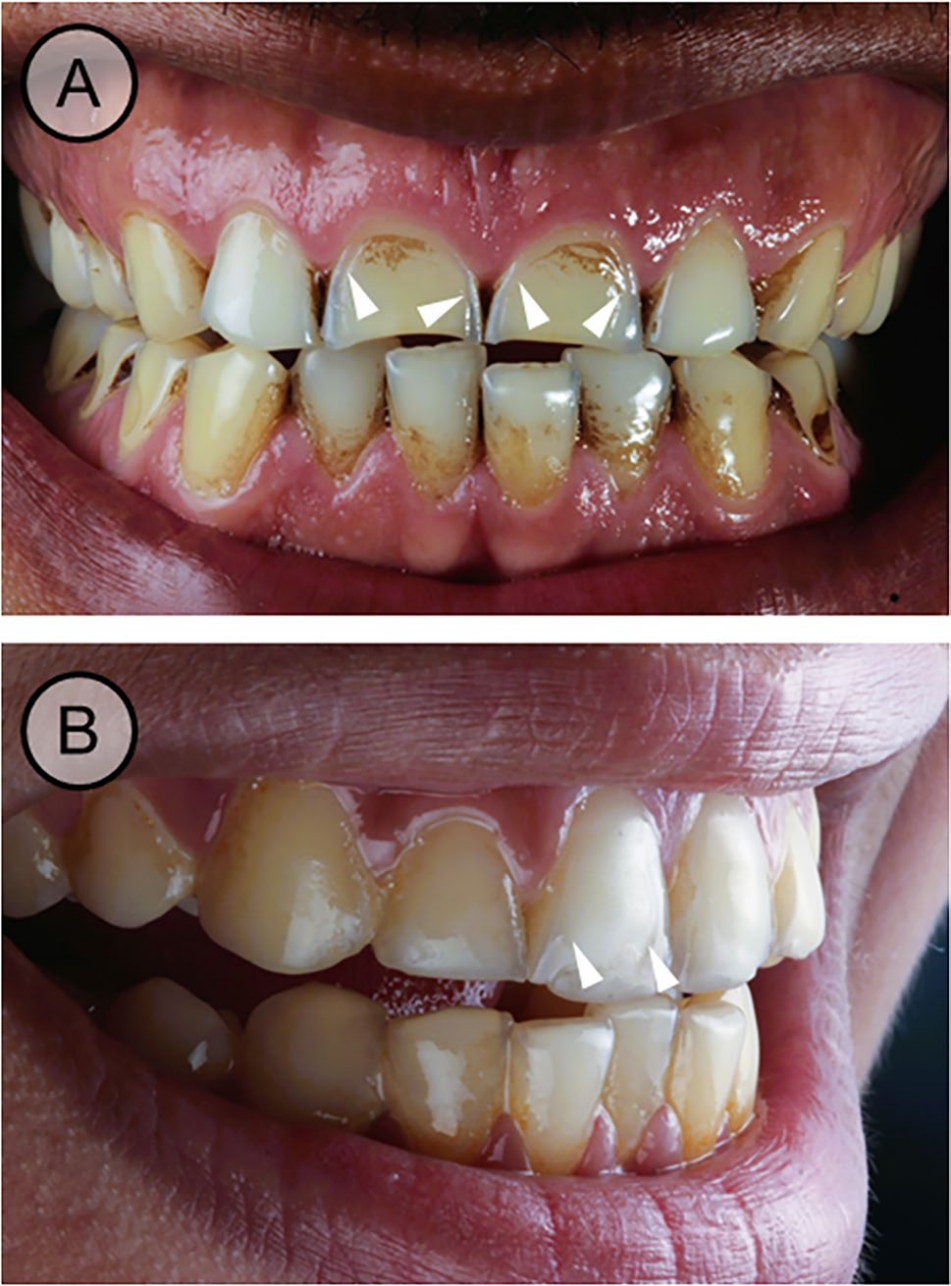
Clinical overview of the erosive lesions on the labial surface of the upper incisors among swimmers. **A** Frontal view of the upper and lower teeth, **B** lateral view of the upper and lower teeth. The white arrows highlight the characteristic veneer preparation-like appearance of the erosive lesions among swimmers

One hundred eighty participants with an average age of 14.7 years were included. Swimmers had significantly more years of practice than non-swimmers (*p* = 0.002). However, there were no significant differences between the training load of both groups per week. Erosive tooth wear was significantly more prevalent in competitive swimmers than non-swimmers (60% and 25.56%, respectively, *p* ˂ 0.001). The mean BEWE score was 1.84 ± 2.09 among swimmers and 0.53 ± 1.10 among non-swimmers, showing a statistically significant difference (*p* ˂ 0.001). Among the competitive swimmers, results of the BEWE evaluation revealed that only 33.3% of participants were at risk of erosion requiring interventional strategies for management. The majority of non-swimmers was at no risk of erosive tooth wear, requiring only routine maintenance and observation. The difference in risk of erosion between the case and control groups was statistically significant (*p* ˂ 0.001) (Table 1). By observation, erosive lesions in competitive swimmers were similar to veneer preparations affecting the labial surfaces of the anterior teeth (Fig. 1).

**Table 1:** Demographic, sports data, and dental erosive lesions prevalence and risk level among study participants

		Swimmers (*n* = 90)	Non-swimmers (Rowers) (*n* = 90)	*p* value
Age: mean ± SD		14.4 ± 1.96	15.1 ± 2.70	0.071
Gender: *n* (%)	Male	59 (65.6%)	57 (63.3%)	0.755
	Female	31 (34.4%)	33 (36.7%)	
Years of practice		8.0 ± 3.14	6.6 ± 2.99	0.002*
Training hrs/week		16.1 ± 6.81	16.2 ± 1.78	0.221
Erosion prevalence: *n* (%)	Yes	54 (60%)	23 (25.56%)	˂ 0.001*
	No	36 (40%)	67 (74.44%)	
BEWE score: mean ±SD		1.8 ± 2.09	0.5 ± 1.10	˂ 0.001*
BEWE risk level: *n* (%)	No risk	60 (66.7%)	83 (92.2%)	˂ 0.001*
	Low	27 (30%)	7 (7.8%)	
	Moderate	2 (2.2%)	(0%)	
	High	1 (1.1%)	(0%)	

*Statistically significant difference at *p* value < 0.05

Regarding salivary parameters among swimmers, there was a tendency for an increase in average salivary pH after the training sessions with a mean percent change of 2.76% ± 4.76 (*p* ˂ 0.001). On the contrary, a significant decrease in salivary pH was detected among non-swimmers after the training sessions. A significant reduction in the salivary flow rate was observed in both groups after practicing with a mean percent change of 9.71% and 13.97% in swimmers and non-swimmers, respectively (Table 2).

**Table 2:** Salivary pH and flow rate (ml/min) changes among swimmers and non-swimmers (Rowers)

Salivary parameters		Swimmers (*n* = 90)	Non-swimmers (Rowers) (*n* = 90)	*p* value
		Mean ± SD	Mean ± SD	
Salivary pH	Before	7.13 ± 0.35	7.32 ± 0.35	˂ 0.001*
	After	7.32 ± 0.37	7.16 ± 0.30	˂ 0.001*
*p* value		˂ 0.001*	˂ 0.001*	
% change		2.76 ± 4.76	– 1.91 ±5.30	˂ 0.001*
Salivary flow rate (ml/min)	Before	1.12 ± 0.38	1.21 ± 0.54	0.375
	After	0.96 ± 0.42	0.92 ± 0.40	0.538
*p* value		˂ 0.001*	˂ 0.001*	
% change		– 9.71 ± 34.99	– 13.97 ± 49.71	˂ 0.001*

*Statistically significant difference at *p* value < 0.05

**Fig. 2:**
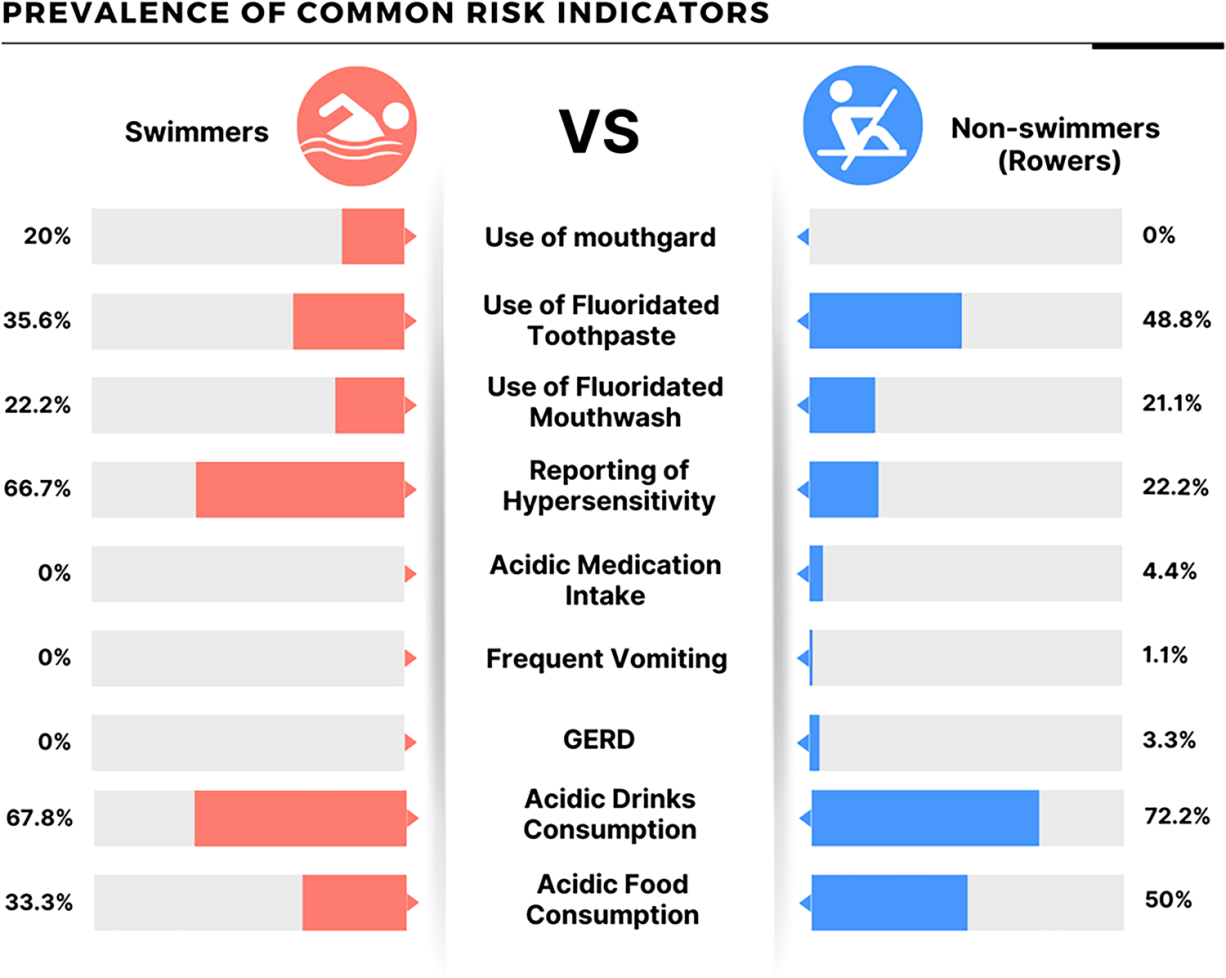
Prevalence of common risk indicators for erosion among swimmers and non-swimmers (Rowers)

The prevalence of several common risk indicators for erosive lesions is illustrated in Fig. 2. Swimmers consumed significantly less acidic food than non-swimmers (33.3% and 50%, respectively, *p* = 0.023). The majority of both swimmers and non-swimmers frequently consumed acidic drinks. Among the non-swimmers, 3.3% reported having medical conditions, 1.1% suffered from frequent vomiting, and 4.4% consumed acidic medications, whereas none of the swimmers reported having any medical conditions or consuming acidic medications. Hypersensitivity was reported by two-thirds of competitive swimmers compared to less than one-third of non-swimmers (*p* ˂ 0.001). With regards to tooth brushing and the use of fluoridated mouthwash, both groups did not differ significantly (*p* = 0.194 and 0.856, respectively). Among all swimmers, only 20% used mouthguards while none of the non-swimmers used mouthguards as protective measures against erosion.

The multivariable logistic regression model revealed that swimmers had a two times higher risk of developing erosive lesions than non-swimmers (AOR = 2.03, 95% CI = 1.78–5.28, *p* = 0.048). Higher odds were significantly related to years of practice (AOR = 1.24, 95% CI = 1.08–1.44, *p* = 0.003). The presence of hypersensitivity and regular consumption of acidic drinks were significant predictors, showing higher odds of erosion AOR = 15.36 and 3.06, respectively, with 95% CI = 6.41–36.84 and 1.15–8.16, respectively, where *p* ˂ 0.001 and *p* = 0.026, respectively. Lower odds for erosive lesions were presented with the use of mouthguards, fluoridated toothpaste, and rinse. However, this association was found to be statistically insignificant (*p* = 0.196, 0.346, 0.636, respectively) (Table 3). With reference to swimming pool water quality, the mean pH level was decreased in all swimming pools throughout the 7 days of assessment. The highest reported mean pH was 7.43 ± 0.42 while the lowest pH was 3.24 ± 0.21 indicating highly acidic swimming pool water.

**Table 3:** Multivariable logistic regression analysis for risk factors associated with erosive tooth wear

	Unadjusted model			Adjusted model		
	UOR	95% CI	*p* value	AOR	95% CI	*p* value
Swimmers vs non-swimmers (Rowers)	4.37	2.32–8.24	˂ 0.001*	2.03	1.78–5.28	0.048*
Years of practice	1.27	1.14–1.42	˂ 0.001*	1.24	1.08–1.44	0.003*
Positive self-reported hypersensitivity	19.52	9.13–41.71	˂ 0.001*	15.36	6.41–36.84	˂ 0.001*
Regular consumption of acidic drinks	2.58	1.28–5.20	0.008*	3.06	1.15–8.16	0.026*
Use of mouth guard	0.84	0.31–2.27	0.725	0.39	0.09–1.63	0.196
Use of fluoridated toothpaste	0.81	0.41–1.60	0.543	0.61	0.21–1.71	0.346
Use of fluoridated mouth rinse	0.91	0.44–1.87	0.803	0.78	0.28–2.19	0.636

*Statistically significant difference at *p* value < 0.05; *UOR* unadjusted odds ratio, *AOR* adjusted odds ratio, *CI* confidence interval

## Discussion

Erosive tooth wear is among the most imperceptible problems to still be considered by dental professionals. Considerable periods of practice time are required by competitive swimmers to improve their swimming skills. Therefore, cumulative exposure to swimming pool water may put them at risk for erosive tooth wear [22]. The study included a cross-sectional sample of 180 athletes, aged from 11 to 25 years. The reason for the selected age of eleven is that it is considered by swimming federations as the age of competition [23]. This age also allowed for the examination of the permanent teeth that have already erupted and been exposed to numerous dental erosion predisposing factors.

According to the study’s results, erosive lesions were more prevalent and severe among the competitive swimmers which agrees with previous studies [16, 17, 24] where prevalence among swimmers ranged from 25.81 to 39% compared to their controls. This may be attributed to swimming pools’ low pH, which may decrease below the salivary pH, leading to erosive lesions [25]. Lack of knowledge about erosive tooth wear and its preventive measures among the swimmers could have also aggravated the condition. This can be confirmed by the findings that only 20% used a mouthguard as a preventive appliance against erosion. Further, the veneer pattern of dental erosive lesions might be explained by the presence of an acquired pellicle layer which acts as a protective barrier against acid attacks. This might be supported by the fact that the salivary pellicle is thicker labially in the anterior teeth. Moreover, most of the pellicle proteins are obtained from salivary glands and originate from crevicular fluid and saliva. Therefore, it has been assumed that the labial surface is protected, especially near the cervical area where proteins are partially derived from crevicular fluid [15] [26].

On the contrary, salivary pH among swimmers showed a significant increase after training by 2.76% while it decreased by 1.91% among the non-swimmers group. However, changes in pH values, in both groups before and after exercising, were still within normal physiological range [21]. Swimmers were not completely restricted from water consumption during their training sessions, which maintains adequate hydration and buffers against the reduction in the pH level. Similarly, studies assessing the rehydration effects in young athletes reported that water consumption suppresses the decrease of salivary pH [27, 28]. In contrast, non-swimmers’ salivary pH tends to drop after training. This is because large levels of carbon dioxide are transported from the blood into saliva, which subsequently lowers the salivary pH [27].

Another important finding in the present study is that, after exercise, the stimulated salivary flow rate was reduced among both groups. This may be attributed to the effect of intense exercise which increases sympathetic activity. The neural control of the autonomic nervous system causes vasoconstriction of the blood vessels supplying the salivary glands resulting in decreased salivary excretion [29]. Reduction of the salivary flow rate may be also a consequence of dehydration due to sweating and fluid restriction. Similarly, several studies showed a reduction in salivary flow rate after different endurance exercises [25, 30].

The multivariable regression model showed that being a swimming athlete significantly increased the risk of erosion 2.03 times more than the non-swimmers. This was in agreement with Centerwall et al. [16], who reported that competitive swimming increased the odds of erosion by 7.2 times. Competitive swimmers were at higher risk of erosion because they spend prolonged time, each week, in the swimming pools. It has been reported that swimmers in a swimming pool were about four to seven times more likely to report swallowing at least a teaspoon of water which increases direct contact with the chlorinated water. This put the swimmers at risk of developing more erosive lesions compared to non-swimmers who were not involved in any activity that required exposing their teeth to the swimming pool water.

The presence of erosive tooth wear was found to have a significant association with the number of years of practice. Each additional year of practice was associated with a 1.24 times higher risk of experiencing more erosive lesions. This finding suggests that erosive lesions are a result of prolonged exposure to risk factors. Since competitive swimmers had, on average, more years of practice than the non-swimmers this increased the likelihood of erosive tooth wear prevalence among them. These results are consistent with a previous study [31] that confirmed that swimmers who practiced for 10 years or more were 3 times at risk than those who swam for less than 10 years.

Dental hypersensitivity was a significant predictor of the prevalence of erosion since it was reported by more than half of the participants. This confirms other studies where competitive swimmers were suffering from severe hypersensitivity, especially in the maxillary and mandibular anterior regions as they are the most commonly affected [31, 32]. This reflects cumulative abusive exposure to acidic factors as in a chlorinated swimming pool environment. Additionally, the results revealed that intake of acidic drinks on a daily basis significantly increased the odds of erosion. Since the consumption of acidic drinks was reported by a high percentage of swimmers, this may add more risk for erosion among them as dietary acids have been associated with erosive tooth wear and hypersensitivity [33].

In the present study, the use of a mouthguard was found to be a protective factor (AOR = 0.39). This appliance prevents direct contact between teeth and acid solutions. Furthermore, it was suggested that lining the mouth guard with any alkaline agents can help neutralize the acidic substance pooling inside it [34]. The use of fluoridated mouth rinses and toothpaste both decreased the odds of erosion (AOR = 0.61 and 0.78, respectively). It has been proven that fluoride-containing products act as protective agents against tooth erosion, and this is attributed to the formation of calcium fluoride-like deposits (CaF2) which reduces the acid-enamel contact. However, this preventive action is limited due to low to moderate fluoride concentrations in commercially available products [35]. This may raise the idea that swimmers may require more potent fluoride products with higher concentrations to provide optimum protection against acid attacks.

One of the study limitations was assessing the effect of acidic food and drinks as well as self-reported hypersensitivity solely via questionnaire may not provide accurate data and be subjected to reporting and recall bias. Another limitation was that within its cross-sectional design, causality is inferred but not proved relative to all the previously mentioned risk indicators of erosive tooth wear which would definitely require further longitudinal investigation. Including a matched control group, on the other hand, is considered a point of strength for this study. It supports the results and strengthens the validity of the detected associations. Furthermore, assessing the salivary parameters for the swimmers’ group is foundational information that added more strength to the investigation since only a few studies were found to evaluate salivary changes among swimmers.

## Conclusions

The findings of the current study suggest that individuals engaged in competitive swimming face a greater susceptibility to the development of erosive lesions. Moreover, an increase in the years of practice and regular consumption of acidic drinks was associated with higher odds of developing erosive lesions. Competitive swimmers exhibited a notable increase in salivary pH subsequent to their training sessions in contrast to the non-swimmers (rowers), despite recording a simultaneous decline in salivary flow rate. The use of mouthguards, fluoridated toothpaste, and rinse were found to reduce the likelihood of erosive tooth wear. These factors may possess the potential to play a pivotal role in addressing this issue and should be incorporated into a comprehensive approach toward promoting the oral health of young athletes.

## Data Availability

The datasets analyzed during the current study are not publicly available; however, they can be available from the corresponding author upon reasonable request.

## Abbreviations

*CDC*: Centers for Disease Control and Prevention
*RWIs*: Recreational Water Illnesses
*BEWE*: Basic Erosive Wear Examination
*DEWRA*: Dental Erosive Wear Risk Assessment
